# Book Reviews

**Published:** 1894-04

**Authors:** 


### *BOOK REVIEWS.*

---

The National Dispensatory—Containing Natural History, Chemistry, Pharmacy, Actions and Uses of Medicines, Including those recognized in the Pharmacopœias of the United States, Great Britain and Germany, with numerous references to the French Codex by Professors Alfred Stillé, M. D., LL. D., John M. Maisch, Phar. D., Charles Caspari, Jr., Ph. G., and Hy. C. C. Maisch, Ph. G., Ph. D. Fifth Edition. Lea Bros. & Co., Philadelphia, 1894.

The first edition of this work appeared in 1879, and that five editions should have been required in the short space of fifteen years, is of itself evidence of the high appreciation the medical and pharmaceutical professions hold for it.

This edition is based on the seventh decennial revision of the United States Pharmacopœia, but it includes all important medicines in use, whether "official" or not. One of the striking characteristics of this work is its authoritative accuracy and the concise and convenient arrangement of desired information, together with the excellent judgment shown in excluding all obsolete matter.

This book is the embodiment of the latest and ripest knowledge of that pharmaceutical savant, John M. Maisch, who had nearly completed his portion of the work when death removed him from the scene of his labors. The work was finished by Professor Charles Caspari, Jr., of the Maryland College of Pharmacy, assisted by H. C. C. Maisch, Ph.D., son of the late Professor Maisch, and reflects much credit on them. While it will be a saving to some, we almost regret the translation of the metric system into the old, as it may prevent in some instances the use of the more scientific decimal system.

The therapeutical department, under the able management of Professor Alfred Stillé, M. D., has been brought well up to date and includes critical statements of all the newer remedies. A very valuable and suggestive "Therapeutical Index" has been arranged, giving practical suggestions for the various diseases, all arranged in alphabetical order. This will prove almost invaluable to the physician as a ready and reliable reference in the treatment of various diseases and be of great assistance in making his prescriptions. This, together with the general index, contains the immense number of 25,000 references. The new Dispensatory contains about 100 pages more than the last and gives many new tables and lists of tests and descriptions of new processes, with elegant illustrations that makes the work of inestimable value to both the pharmacist and physician. H. R. S.

---

AN AMERICAN TEXT-BOOK OF THE THEORY AND PRACTICE OF MEDICINE. Edited by William Pepper, M. D., LL. D., Professor of the Theory and Practice of Medicine in the University of Pennsylvania. In two volumes, Volume II. illustrated. W. B. Saunders, Publisher, 925 Walnut Street, Philadelphia.

The first volume of this excellent work has already been reviewed in these pages, and it was our pleasure to give it a hearty recommendation. The second volume is perhaps even better than the first, and the completed work makes a treatise on the practice of medicine which, for practical utility to the reader, has not a superior in the language. This is a composite work, representing and containing the views and experience of several of the most prominent American teachers and practitioners. This second volume begins with an excellent chapter by Dr. Welch, of Johns Hopkins University, on the Biology of Bacteria, Infection and Immunity. Dr. Henry W. Lyman, of Chicago, contributes the chapters on Diathetic Diseases, Rickets, Lithiasis, Diabetes, Rheumatism, etc. Diseases of the Heart, Blood-vessels, and Diseases of the Intestines are described by the editor, Dr. Pepper. Dr. Francis Delafield, of New York, furnishes the chapters on the Lungs and Kidneys. Other contributors are Drs. William Osler, of Baltimore; Reginald H. Fritz, of Boston; James C. Wilson and James W. Holland, of Philadelphia. The fact that this work has been prepared by different writers has enabled them to bring it absolutely up to date. The use of the latest drugs and the latest methods of treatment receive proper attention whenever necessary. This work is by far the best of the American Text-book Series that has ever issued from the press of Mr. Saunders.

---

REFERENCE HAND-BOOK OF MEDICAL SCIENCES. Vol. IX. Supplement. By various writers. Edited by Albert H. Buck, M. D., New York. William Wood & Co., New York.

This supplement to the Reference Hand-Book series, first published a few years ago is the outcome of a decision to bring this valuable work fully up to date. The original volumes have proven their great usefulness, and have found places in many libraries. But so great has been the progress along certain lines of medicine, such as Pathology, Surgery and Therapeutics, that it has been deemed wise to add another volume which would contain the main features of our more recently acquired knowledge. In addition, a number of entirely new articles have been introduced which were not discussed at all in the original work. One has but to read the sections on Cholera, Influenza, Intestinal Surgery, Tuberculosis, Wound Treatment, etc., to see how well the work of revision has been executed. The last volume fully merits all the praise which has been given its predecessors. Those who have the latter should possess the former by all means.

---

THE MODERN CLIMATIC TREATMENT OF INVALIDS WITH PULMONARY CONSUMPTION IN SOUTHERN CALIFORNIA. By P. C. Remondino, M. D., San Diego, California. Physicians' Leisure Library Series. Twenty-five cents. George S. Davis, Detroit, Michigan.

This little book is a description of the peculiar climatic and hygienic conditions which exist in Southern California, with special reference to the treatment of invalids, not only with pulmonary consumption but also with diseases of the kidneys, rheumatism, malaria, etc. The author attempts to show for that section "that a well man will stand less chance of becoming sick here, and that an invalid will, in the average, live longer and more comfortably, and with the greatest probable chances of an ultimate recovery, than in any other portion of the United States."

---

THE PHYISCIAN'S WIFE. By Ellen M. Firebaugh. Illustrated with forty-four engravings of sketches from life. The F. A. Davis Company, Philadelphia.

The author of this little book is the wife of a practicing physician in Illinois, and gives ample evidence of being well acquainted with the country doctor's mode of life. The book really had its origin in a perusal of Dr. Cathill's admirable volume on "The Physician Himself," and while Mrs. Firebaugh's effort falls far short of her model, she has succeeded in giving us some good pictures from the life of the country practitioner. Many of her descriptions are rather trivial, and she has failed to touch upon other subjects which would find an appropriate place in a work of this sort. But withal, a very readable little work.

ANNUAL OF THE UNIVERSAL MEDICAL SCIENCES. Edited by Charles E. Sajous, M. D. Five volumes. 1893. F. A. Davis Company, Philadelphia.

This work is well known to our readers, many of whom have used the preceding issues. It is a work of great magnitude and value. The editor has had the active assistance of seventy associates and the co-operation of over two hundred corresponding editors and collaborators. The present series fully sustains the merit of previous issues, and the work continues to furnish a report of the progress of the general medical and sanitary sciences throughout the world. We cheerfully recommend the 1893 series to all who wish to keep thoroughly informed in the most recent advancements in our art.

